# Experience with the core curricular elements for international emergency medicine fellowships

**DOI:** 10.1186/1865-1380-6-10

**Published:** 2013-04-15

**Authors:** David I Beran, Jennifer Avegno

**Affiliations:** 1 Department of Medicine, Section of Emergency Medicine, Louisiana State University Health Sciences Center, 2020 Gravier St. 7th Floor, New Orleans, LA, 70112, USA; 2 Department of Surgery, Tulane Medical School, Department of Surgery/SL 22, 1430 Tulane Avenue, New Orleans, LA, 70112-2699, USA

**Keywords:** International emergency medicine, Fellowships, International emergency medicine fellowships, Curriculum, International emergency medicine fellowship curriculum

## Abstract

**Background:**

The number of international emergency medicine (IEM) fellowships available in the US has grown dramatically since the inception of subspecialty training in 1994 Bayram et al. (Acad Emerg Med 17:748–757, 2010). These fellowships vary according to their curricular structure, intensity of fellow exposure and requirements for program completion. The variety of fellowship structures may have negative connotations for graduates from its fellowships and reflect upon the translatability of their skill sets.

The recent article “Core Curricular Elements for International Emergency Medicine Fellowships” Alagappan and Holliman (Emerg Med Clin 23(1):1–10, 2005) was designed as a curricular development tool and enumerates seven foci within the broad field of IEM.

**Objectives:**

The authors of this article describe their experience using this curriculum development tool. Individual experiences in each of the seven categories described in the “Core Curricular Elements” article were identified and undertaken within the typical 2-year training period.

**Discussion:**

A curricular structure is described that integrates exposure to all seven areas along with the Master’s of Public Health (MPH) degree, the clinical component and the academic component thematic to existing fellowships. Benefits of this curriculum include increased exposure to multiple areas of IEM, potential for greater standardization and increased translatability of skill set. Disadvantages include superficial exposure to areas of IEM and potentially decreased travel time.

**Conclusion:**

The result is a plausible curriculum where fellows would gain exposure to more areas of IEM than they may have otherwise while still earning their MPH, working clinical shifts and carrying out academic fellowship requirements. The authors conclude that this structure allows fellowships to continue drawing on their strengths, provides a more well-rounded fellowship experience and increases structure without requiring standardization.

## Introduction

In a 2010 article titled “Core Curricular Elements for International Emergency Medicine Fellowships,” Bayram et al. [1] outlined curricular components for those designing fellowship curricula [2]. The article describes seven different categories of experience that international emergency medicine (IEM) fellows tend to undertake and gives examples and scope of these experiences. We describe our experience applying these categories to curricular design for an IEM fellowship and prove the plausibility of such a design.

## Background

The field of IEM has emerged as a hybrid of emergency medicine (EM) and public health applied to a global scale [3]. The fellowships are diverse in terms of the experiences they afford fellows, their connections and their scope of research [4]. There is potential variability in the IEM fellowship experience both within and across fellowships. Fellows can have diverging experiences with varying lengths of time abroad, in situations of differing intensity and with variable amounts of actual hands-on involvement [4].

What then does it mean to be a graduate of an IEM fellowship? Does it guarantee a certain amount of experience in humanitarian relief or a level of familiarity with intra-organizational communication? Does it translate into practical knowledge of resource allocation in times of disaster or expertise in grant writing? In the current training system, it could mean all of these things. Many graduates will continue their careers in academic emergency medicine and oversee future fellows, residents and medical students who may not have the same academic interests. There is a theoretical advantage to designing training programs that expose fellows to as many aspects of the broad field of IEM as possible.

The core curricular elements article defines seven areas of IEM where experiences tend to be undertaken. We hypothesized that within a typical 2-year fellowship period, fellows could have at least one experience in each of the seven areas and earn a Master’s of Public Health (MPH) degree while carrying out the academic and clinical requirements of the fellowship program. We then combined this hypothesis with common features of existing IEM curricula to design a new curricular structure and set out to execute it.

### The approach to an applied curricular structure

Initially, the Society for Academic Emergency Medicine (SAEM) website was used to compile a list of existing IEM fellowships [5]. The individual fellowship websites were then reviewed for content and fellowship structure. Many programs publish requirements of their curricula online and, from these, major fellowship features and a conclusion regarding the overall organization of fellowships were drawn. It was noted that both 1- and 2-year fellowship programs exist. Since we wanted to explore the plausibility of exposure to all seven areas while earning an MPH, we focused on the curricula of 2-year programs. While earning an MPH is not necessarily a requirement of IEM fellowships, we noted that most fellowships offer one as a part of the fellowship. We chose to require this. The fellowships tend to have two other major components: clinical and academic. Average workloads of each component were then determined and applied to the proposed curriculum. The themes of the fellowships were then woven into the new framework and integrated into the concept of equivalent experiences.

### IEM domains and equivalent experiences

The seven areas described by the core curricular elements article are EM systems, humanitarian relief, disaster management, public health, travel and field medicine, program administration and academic skills. For the purpose of this curriculum, the seven fields were called “domains” of international emergency medicine. Within each domain, many separate experiences are available in the form of real-life scenarios, conferences, symposia, workshops or research. These various experiences were termed “equivalent experiences.” Therefore, there are many possible equivalent experiences within each domain (Table 1). Any fellowship program could make its own list of equivalent experiences for each domain and draw upon the pre-existing strengths and connections of that fellowship. Practically, this means that a fellow will be expected to carry out an experience in each domain not covered by the clinical, academic or MPH components of the fellowship.

**Table 1:** Examples of equivalent experiences

**Domain**	**EM systems**	**Humanitarian relief**	**Disaster medicine**	**Public health**
**Example of equivalent experience**	NAEMSP Medical Direction Course	Humanitarian mission	Work on a DMAT	M.P.H. coursework
	International Ambassadorship through ACEP	HELP/ HELP II course	Volunteer with NGO during disaster	Burnet Institute short courses in public health
	Serve on professional committee (AAEM, ACEP)	Public Health in Complex Emergencies course (PCHE)	ACS DMEP course	WHO communicable disease course
	Active role in int’l organization	Working with NGO	DRLA course or certificate	Harvard International Health System Short course
**Domain**	**Travel and field medicine**	**Program administration**	**Academic skills**	
**Example of equivalent experience**	ASTM&H or RSTM&H Conference	Grant-writing experience	Journal clubs	
	Work in a travel medicine clinic	Attend a grant-writing workshop	Yearly lectures to residents	
	Wilderness Medical Society Course	Curriculum development	Presentation at int’l conference	
	Tropical Med certificate course	Rotation site development	Producing a publishable work	

Explanation of abbreviations read across cells: National Association of Emergency Medical Services Physicians, Disaster Management Assistance Team, American Society of Tropical Medicine and Hygiene/Royal School of Tropical Medicine and Hygiene, American College of Emergency Physicians, Health Emergencies in Large Populations/Health, Ethics, Law and Politics, American Academy of Emergency Physicians, American College of Surgeons Disaster Management & Emergency Preparedness, World Health Organization, Disaster Resilience Leadership Academy at Tulane.

### Clinical requirement

Many fellowships did not publish their requirements or published them in the unit of “shifts,” which could mean any number of hours. Further, some fellowships have different arrangements with fellows where they may be called fellows or expected to act as an attending physician. As there is no unified definition of these terms, an industry average of 6–8 12-h shifts each month was used. These shifts were averaged over the course of 1 year, meaning the fellow had to work approximately 84 12-h shifts in 1 year. This arrangement was made to allow for travel.

### Academic requirements

The academic requirement for the proposed curriculum was also taken as a time average based on the requirements of existing fellowships. Nearly all fellowship websites state that the fellow will assume the responsibilities of an attending physician. This generally translates into teaching during clinical shifts, giving lectures, participating in grand rounds and mentoring responsibilities. Also, participation in journal clubs is a common requirement.

There were several academic requirements made for the proposed curriculum aside from teaching during clinical shift work. Firstly, the fellow is to arrange and present at a monthly journal club for all months the fellow is present. In addition to this, the fellow must lecture in an EM residency conference setting once per year and attend one internationally recognized conference per academic year. Also, the fellow is expected to mentor EM residents in IEM research and assist residents in IEM involvement should they pursue it.

### MPH coursework

MPH coursework is dictated by the associated school of public health. For this program, the school requires its MPH graduates to carry out a public health analysis (PHA), which is equivalent to a thesis. Also, students must complete a culminating experience, which is a hands-on, 200-h public health experience secured and carried out entirely by the student. For the purposes of the proposed curriculum, both the PHA and the culminating experience were required to have an IEM focus, agreed upon independently by the IEM fellowship director and the student’s mentor at the school of public health.

The structure for the new fellowship takes industry averages for its clinical, academic and MPH components. The integration of equivalent experiences derived from the core curricular elements categories is the feature of this curriculum that expands the fellowship scope and the experiences fellows will undertake. The overall structure requires that fellows be exposed to each of the seven areas and may use their MPH or academic components to meet some of these requirements. What areas are not covered through the MPH or academic components must be carried out through predetermined “equivalent experiences.”

As an illustration, if a fellow finished their MPH, wrote their thesis on global positioning systems in times of disaster, carried out their culminating experience in a travel medicine clinic and finished their academic requirement, they would have demonstrated exposure to four domains (public health, disaster medicine, travel and field medicine and academic skills). In this new proposed curriculum, they will still have to undertake an equivalent experience in EM systems, humanitarian relief and program administration in order to meet the curricular requirements.

## Findings

The 2-year period described here began on 1 July 2010 and ended on 30 June 2012.

The clinical requirements of the fellowship were met. The author averaged 7.99 12-h shifts per month in the first year and 7.28 per month in the second year, carried out at an urban level-1 trauma center. The range of hours per month was between 0 and 14 shifts/month for the first year and 0 and 9.75 shifts/month during the second. The clinical requirement of averaging 6–8 12-h shifts each month was therefore satisfied.

The academic requirements of the fellowship were also met. Journal clubs were held regularly, and the author also provided both IEM and EM lectures at the rate of two total lectures/year along with annual attendance of national or international conferences. Also in the realm of academics, the author co-mentored two chief residents and a medical student on their IEM project regarding the ethics of humanitarian aid. This project was presented at the International Conference on Emergency Medicine in Dublin, Ireland, in June 2012 and will subsequently be submitted for publication.

The MPH portion has been satisfied through completion of the requisite coursework. The culminating experience was used to develop a lecture site and rotational experience for future fellows and current residents in Kakinada, India. The PHA describes this curriculum in detail (both a fall largely under the program administration domain). This left four domains (EM Systems, Humanitarian Relief, Disaster Management and Travel/Field Medicine) for the author to complete an equivalent experience during the 2-year period.

For the EM systems domain, the author became the international ambassador to Costa Rica for the American College of Emergency Physicians. This role requires the production of an annual report regarding the state of EM in that country as well as functioning as a liaison between EM physicians in that country and the US. The humanitarian relief domain was satisfied through two experiences: providing healthcare in clinics in and around Jinotega, Nicaragua, as well as through completing the Health Emergencies in Large Populations (HELP) course offered at Johns Hopkins University. Tulane University offers a certificate program through their Disaster Resilience Leadership Academy through which the author took a graduate level course and earned credits satisfying the disaster management requirement. Lastly, certification was earned in Advanced Wilderness Life Support (AWLS) through the Wilderness Medical Society to complete the travel and field medicine domain.

## Discussion

This curriculum has several benefits. Principally, this experience provides structure without requiring standardization. Any new fellowship implementing this structure could draw upon its connections and incorporate them into this framework. Similarly, an existing fellowship could adopt this structure and keep in place its connections and strengths. Given the broad range of what could constitute an equivalent experience, fellowships could retain their diversity while providing more structure to their fellows. This increased structure provides more exposure to more areas of IEM than previously required. As an example, exposure to the ACEP ambassadorship, HELP course, AWLS course or disaster leadership course might not have been gained through a different fellowship structure.

This structure also improves the translatability of the fellow’s skill set. Fellows graduating from this program would be more likely to graduate with certifications, conference or workshop attendance that they might not have otherwise received. This translates into a well-rounded educational experience that benefits not only the fellow, but the fellows, residents and medical students they will likely mentor one day. Further, this is beneficial from the perspective of future employers who may perceive graduates as having a broader range of skills that suits them for a variety of practice settings.

There are several potential disadvantages to this structure as well. Notably, that exposure and proficiency are two separate entities. Having a certification in wilderness medicine does not make one proficient in it and certainly cannot take the place of a wilderness medicine fellowship or an IEM fellowship where the fellow focuses primarily on medicine in austere environments. By the general nature of these programs, fellows will spend disproportionately more time in one or two domains than the others. Using this example, developing a lecture site is much more time consuming and intense than a 3-day AWLS course.

Another issue with this structure is that equivalent experiences are not necessarily equivalent. A 3-day disaster medicine course cannot really be equivalent to a 3-week HELP course in terms of intensity or scope. This would have to be addressed by fellowship directors a priori. Fellowships could make their equivalent experiences more equivalent by deciding upon a time commitment or CME minima beforehand.

Also, many experiences do not neatly fit into only one category. A fellow traveling to post-earthquake Haiti could easily and correctly argue that their experience covered humanitarian relief, disaster medicine, and travel and field medicine domain requirements Fellowship directors would have to categorize experiences with fellows beforehand as being in one domain or another to avoid any discrepancies after the experience has been carried out.

There is also the potential disadvantage of decreased travel time while fellows carry out equivalent experiences. Fellows who have different academic interests than the authors may have a different experience.

## Conclusion

The authors demonstrate the plausibility of a curriculum for an IEM fellowship that provides increased structure for fellows. The benefit of this structure compared to existing fellowships will have to be demonstrated through the implementation of this fellowship, graduation of fellows and comparing their experiences to fellows of other curricula. Despite the potential disadvantages of this structure, the authors contend that increased structure and exposure to more areas of IEM will lead to an increased translatability of the fellow’s skill set and is a positive option for fellowship structure.

## Competing interests

The authors declare that they have no competing interests.

## Authors’ contributions

DB carried out the experiences mentioned in the manuscript and drafted the initial manuscript. DB conducted literature and online review of fellowship programs and developed the list of equivalent experiences. JA edited, reviewed and assisted with research supporting the manuscript. Both authors read and approved the final manuscript.
